# Early Life Fructose Exposure and Its Implications for Long-Term Cardiometabolic Health in Offspring

**DOI:** 10.3390/nu8110685

**Published:** 2016-11-01

**Authors:** Jia Zheng, Qianyun Feng, Qian Zhang, Tong Wang, Xinhua Xiao

**Affiliations:** 1 Department of Endocrinology, Key Laboratory of Endocrinology, Ministry of Health, Peking Union Medical College Hospital, Diabetes Research Center of Chinese Academy of Medical Sciences & Peking Union Medical College, Beijing 100730, China; zhengjiapumc@163.com (J.Z.); rubiacordifolia@yahoo.com (Q.Z.); tongtong0716@sina.com (T.W.); 2 Department of Pediatrics, The Second Teaching Hospital of Tianjin University of Traditional Chinese Medicine, Tianjin 300193, China; fengqianyun@yahoo.com

**Keywords:** early life, fructose, sugar-sweetened beverages, cardiometabolic health, offspring

## Abstract

It has become increasingly clear that maternal nutrition can strongly influence the susceptibility of adult offspring to cardiometabolic disease. For decades, it has been thought that excessive intake of fructose, such as sugar-sweetened beverages and foods, has been linked to increased risk of obesity, type 2 diabetes, and cardiovascular disease in various populations. These deleterious effects of excess fructose consumption in adults are well researched, but limited data are available on the long-term effects of high fructose exposure during gestation, lactation, and infancy. This review aims to examine the evidence linking early life fructose exposure during critical periods of development and its implications for long-term cardiometabolic health in offspring.

## 1. Introduction

The prevalence of obesity and type 2 diabetes are increasing dramatically throughout the world, now considered a pandemic non-communicable disease. In 2015, the International Diabetes Federation estimated that 415 million people worldwide have diabetes, and the number will rise to 642 million by 2040, implying that one in eleven adults will have diabetes. Moreover, one in seven births is affected by gestational diabetes [1]. As such, type 2 diabetes yields enormous tolls at individual, public health, and economic levels.

In recent years, it has become increasingly clear that susceptibility to obesity and type 2 diabetes is strongly influenced by exposure to an adverse early life development environment during pregnancy and postnatal life. A combination of human epidemiology studies and rodent studies has clearly established that maternal environment—especially nutrition during pregnancy and the postnatal period—are critical factors influencing the development of cardiometabolic disease, such as obesity, type 2 diabetes, and cardiovascular diseases in offspring [2,3,4]. Growing numbers of clinical and animal studies suggest that maternal consumption of a diet high in fat and other potential nutrients promotes obesity and increased metabolic risk in offspring. However, little is known about the effects of fructose exposure during early life development.

## 2. An Overview of Fructose

### 2.1. Consumption of Fructose Is Increasing

Fructose, or fruit sugar, is a simple ketonic monosaccharide found in many plants. It is one of the three dietary monosaccharides, and can be absorbed directly into the bloodstream. Fructose is widely used commercially in foods and beverages, due to its low cost and high relative sweetness. Fructose is the sweetest of all naturally-occurring carbohydrates; however, we rarely consume fructose in isolation. The major source of fructose in the diet comes from fructose-containing sugars, such as sucrose and high fructose corn syrup (HFCS) [5]. A national survey in the United States showed that the mean intake of total fructose increased from 8.1% in 1978 to 9.1% in 2004 as a percentage of total energy. It is important to note that this increase was greater in adolescents and young adults [6]. The intake of refined sugar—particularly HFCS—has increased from a yearly estimate of 8.1 kg/person at the beginning of the nineteenth century to a current estimate of 65 kg/person [7]. Sugar-sweetened beverages (SSBs) are the greatest source of fructose-containing sugars in the diet, and the consumption of SSBs shows a steady increase in both children and adults [8]. The National Health and Nutrition Examination Survey showed that one-half of the population consumes SSBs on any given day, and 25% consumes at least 200 kcal in United States [9]. It is noticeable that fructose was primarily from artificially sweetened beverages and SSBs, but not from naturally occurring fructose in fruits.

### 2.2. Adverse Metabolic Effects of Fructose

The consumption of fructose has become a hot topic, due to its multiple metabolic effects [10]. For decades, it has been thought that excessive intake of fructose from SSBs and foods has been linked to increased risk of obesity, type 2 diabetes, and cardiovascular disease in various populations [11]. One large clinical study showed a close parallel between the rise in HFCS intake and the obesity and diabetes epidemics in the United States [12]. Excess fructose consumption has been demonstrated to be a risk factor of insulin resistance [13], elevated low-density lipoprotein cholesterol (LDL-c), and triglycerides [14], leading to obesity, type 2 diabetes, and cardiovascular disease [15]. The Nurses’ Health Study cohort study showed that women consuming one or more sugar-sweetened soft drinks per day had an 83% greater risk of developing type 2 diabetes mellitus over the course of eight years compared with those who consumed less than one of these beverages per month [16]. It also indicated that a higher level of SSB intake was found to increase the risk of developing nonfatal myocardial infarction and fatal coronary heart disease, and women who consumed ≥2 SSBs per day had a 35% greater risk of coronary heart disease, compared to infrequent consumers [17]. One recent meta-analysis of nine prospective cohort studies with 308,420 participants, conducted in the USA, Japan, Sweden, and Singapore found a greater risk of myocardial infarction and stroke with incremental increase in the consumption of SSBs [18]. Therefore, widespread increase in dietary fructose consumption is associated with the development of chronic cardiometabolic disorders.

## 3. Early Life Fructose Exposure and Long-Term Cardiometabolic Health

### 3.1. Implications of Human Studies

The deleterious effects of excess fructose consumption in adults are well researched, but limited data are available on the long-term effects of high fructose exposure during gestation, lactation, and infancy. More importantly, emerging research suggests that fructose consumption by both mothers and their offspring during these stages of early life can lead to persistent metabolic dysfunction. It is common sense that fresh fruit and vegetable intake during pregnancy has multiple benefits to the mothers and babies. One clinical cohort study of pregnant women conducted by the Norwegian Institute of Public Health found that intakes of foods high in natural sugars (such as fresh and dried fruits) are associated with decreased risk of preeclampsia [19,20]. However, because fructose was mainly from artificially sweetened beverages and SSBs and not from natural fruit, most pregnant women are exposed to the same artificially sweetened foods and beverages as the general non-pregnant population. It reported that added sugar represents 14% of the energy intake in diets consumed by pregnant women [21]. Little clinical research exists addressing the effect of excessive fructose during pregnancy. One large prospective cohort study of 60,761 pregnant women showed that high intake of both artificially sweetened beverages and SSBs during pregnancy were associated with increased risks of preterm delivery [22]. Therefore, high intake of fructose from artificially sweetened beverages and SSBs will impact pregnancy outcomes.

### 3.2. Implications of Rodent Experiments

Some rodent experiments have also demonstrated that excessive fructose consumption during early development can increase the incidence of metabolic disorders Studies in rats have shown that the adult male offspring suckled by mothers consuming an iso-caloric fructose-rich diet during lactation displayed increased body weight and food intake, enhanced leptinemia, and impaired insulin sensitivity, with decreased hypothalamic ob-Rb gene expression and STAT-3 phosphorylation [23]. In rats, dams fed a high fructose (20%) solution during pregnancy and lactation displayed sex-specific effects on placental growth and fetal and neonatal metabolic profiles [24]. Maternal fructose intake significantly elevated circulating plasma fructose and leptin levels in female fetuses. By postnatal day 10, both male and female neonates born to fructose-fed mothers showed high circulating fructose and insulin levels, as well as increased leptin content [24]. Additional studies showed that dams with fed 100 g/L fructose ate more food and drank less water. Moreover, the offspring of fructose-fed dams had almost double the fasting insulin levels at weaning compared with the offspring of glucose-fed dams [25].

Further experiments revealed that a maternal 60% fructose diet led to increased serum triglycerides, free fatty acids, and insulin in offspring at 23 weeks old. This was concomitant with elevated increased expression of carnitine palmitoyltransferase (CPT1a) and acetyl-coenzyme A carboxylase beta (ACC2), and decreased expression of peroxisome proliferatoractivated receptor-α (PPARα) and PPAR-gamma coactivator 1-α (PGC1-α) [26]. A recent study showed that maternal consumption of a high-fructose diet leads to the developmental programming of adverse cardiometabolic health in offspring at 1 year of age, including obesity, hypertension, insulin resistance, increased liver fat infiltrates, and visceral adipose tissue [27]. Another recent study found that male offspring exposed to a maternal fructose-rich diet during pregnancy developed severe hyperglycemia, hypertriglyceridemia, hyperleptinemia, and augmented adipose tissue mass with hypertrophic adipocytes [28]. Gray et al. showed that excess fructose consumption before and during pregnancy lead to a marked skew in the secondary sex ratio and reduced fertility, reflected as a 50% reduction in preimplantation and term litter size [29]. They further found that increased fructose in the maternal diet had lasting effects on offspring cardiovascular function, including hypertension, heart rate, and relative non-dipping of nocturnal pressure that was sex-dependent and related to the offspring’s stress–response axis. Up-regulation of vasoconstrictor, anti-natriuretic, or diminished vasodilatory pathways may be causal [30]. Tain et al. found that maternal high-fructose diet caused increases in blood pressure in the 12-week-old offspring, and melatonin therapy blunted the high-fructose-induced programmed hypertension and increased nitric oxide level in the kidney [31]. They further showed that aliskiren administration prevented high-fructose-induced programmed hypertension in both sexes of adult offspring, which increased angiotensin-converting enzyme 2 and angiotensin (1–7) receptor (MAS) protein levels in female kidneys [32]. Together, the above studies (summarized in Table 1) suggest that excessive fructose exposure during fetal and early postnatal development increases the susceptibility to hypertension, hypertriglyceridemia, insulin resistance, and possibly other metabolic disturbances later in life.

### 3.3. Potential Mechanisms of Early Life Fructose Exposure and Cardiometabolic Health

Gestation and early postnatal life is a critical time window that can affect the growth and development of offspring. It is widely accepted that maternal and postnatal nutrition status is a key determinant of offspring health. The Developmental Origins of Health and Disease (DOHaD) hypothesis proposes that exposures during early life play a critical role in determining the risk of developing metabolic diseases in adulthood, and is also known as the “fetal programming hypothesis” [33]. Although little information is available about the mechanisms between early life fructose exposure and cardiometabolic health, we speculate that “metabolic programming” is the underlying mechanism, because it can link maternal nutrition and metabolic health in offspring.

**Figure 1:**
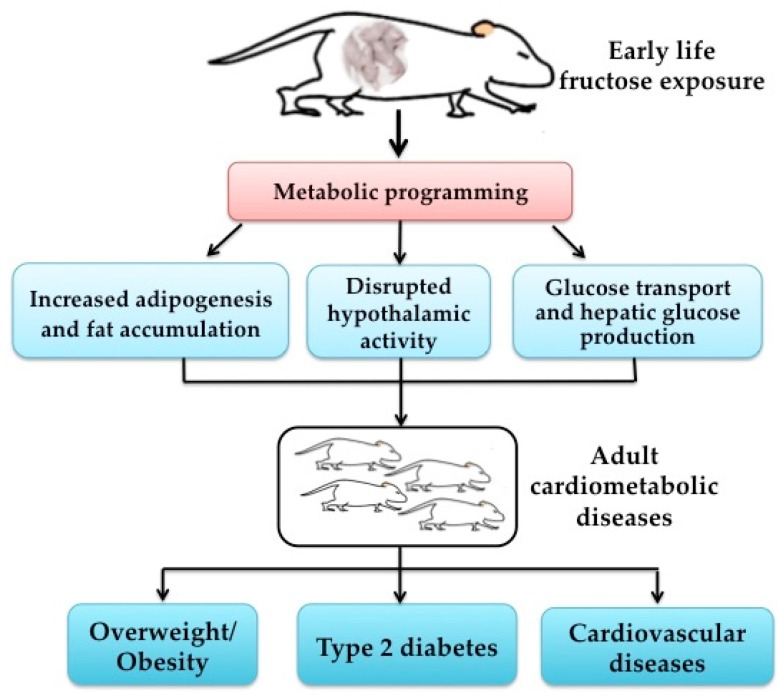
Early life fructose exposure and long-term cardiometabolic health.

Several potential points could explain the adverse effects of high fructose exposure during these periods, which are summarized in Figure 1. First, fructose can bypass the main rate-limiting step of glycolysis at the level of phosphofructokinase and provide lipogenic substrates for conversion to fatty acids and triglycerides as well as transcription factors and enzymes involved in lipogenesis, including acetyl-coenzyme A carboxylase and sterol regulatory element binding protein 1c; thus, it can lead to increased hepatic de novo lipogenesis [5]. Second, a rodent study showed that a maternal fructose-rich diet during lactation decreased hypothalamic sensitivity to exogenous leptin, enhanced food intake, and decreased several anorexigenic signals (e.g., corticotropin-releasing hormone, thyrotropin-releasing hormone, cocaine- and amphetamine-regulated transcript, proopiomelanocortin) in offspring [23]. Third, fructose is transported passively across membranes by a member of the facilitative glucose transporter (GLUT) family, named GLUT5 [34]. David et al. showed that GLUT5 expression and function in weaning and post-weaning rats can be markedly enhanced in vivo by the consumption of high-fructose diets [35]. However, it is still unclear whether the impacts on the offspring are the direct effects of fructose transfer through the placenta or the mother’s milk, or due to adaptive responses to altered maternal metabolism. Thus, further studies are urgently warranted to clarify the underlying mechanisms.

## 4. Conclusions

In summary, pregnancy and early postnatal life are the critical periods of growth and development, and are sensitive to the environment. One important point that should be taken into consideration is that early life fructose exposure may determine the susceptibility of long-term metabolic diseases in offspring. However, limited data suggest that the offspring would be protected from these well-known adverse effects during early life. More intervention studies are necessary to explore the beneficial measures. Moreover, little information is available regarding which period is more critical to determine the risks of cardiometabolic diseases in offspring. Further investigations are imperative to determine the effects of excess fructose consumption during critical periods of gestation, lactation, and early postnatal period. The increasing rates of obesity, prediabetes, and diabetes in individuals of reproductive age can initiate a vicious cycle, propagating risk to subsequent generations. A better understanding of the role and mechanism of early life fructose exposure and metabolic health can provide critical implications for the early prevention of obesity and type 2 diabetes, and ensure a healthier future for subsequent generations.

## Figures and Tables

**Table 1:** Summary of early life fructose exposure and cardiometabolic health in rodents.

Fructose Exposure	Species	Age	Metabolic Disorders	Potential Mechanism	Reference
Maternal iso-caloric 10% fructose rich diet during lactation	Sprague Dawley rats	Between 49–60 days	Increased body weight and food intake, enhanced leptinemia, and impaired insulin sensitivity	Disrupted hypothalamic activity: decreased hypothalamic ob-Rb gene expression and STAT-3 phosphorylation	Alzamendi et al. [23]
Maternal 20% of caloric intake from fructose from day 1 of pregnancy until postnatal day 10	Wistar rats	Embryonic day 21 and postnatal day 10	Elevated circulating plasma fructose, insulin, and leptin levels	Placental fructose sensitivity and transfer: glucose transporter 5 and IGF-1	Vickers et al. [24]
Maternal 100 g/L fructose water during pregnancy	Sprague Dawley rats	Postweaning day 5	Hyperglycemia and hyperinsulinemia	Elevated phosphoenolpyruvate carboxykinase	Rawana et al. [25]
Maternal 60% fructose throughout pregnancy and lactation	Sprague Dawley rats	14–23 weeks old	Increased serum triglycerides, free fatty acids, and insulin	Increased expression of ACC2 and CPT1α, and decreased expression of PPARα and PGC1-α	Ching et al. [26]
Maternal 10% fructose during pregnancy	C57BL/6J mouse	1 year old	Hypertension, insulin resistance, and obesity	Increased expression of PTP1B and JNK	Saad et al. [27]
Maternal 10% fructose during pregnancy	Sprague Dawley rats	60 days	Hyperglycemia, hypertriglyceridemia, and hyperleptinemia	Reduced adipocyte precursor cells number	Alzamendi et al. [28]
Maternal 10% fructose during before conception and during the mating period	Sprague Dawley rats	At day 20 gestation	Growth, fertility, sex ratio, and birth order	Glycolyzable monosaccharide on the maternal ovary and/or ovulated oocyte	Gray et al. [29]
Maternal 10% fructose before and during gestation and through lactation	Sprague Dawley rats	9 to 14 weeks of age	Hypertension	Vasoconstrictor, anti-natriuretic, or diminished vasodilatory pathways	Gray et al. [30]
60% fructose throughout pregnancy and lactation	Sprague Dawley rats	12 weeks of age	Hypertension	Nitric oxide and arachidonic acid metabolites	Tain et al. [31]
60% fructose throughout pregnancy and lactation	Sprague Dawley rats	12 weeks of age	Hypertension	ACE and MAS	Hsu et al. [32]

STAT-3: signal transducer and activator of transcription-3; IGF-1: insulin-like growth factors-1; ACC2: acetyl-coenzyme A carboxylase beta; CPT1a: carnitine palmitoyltransferase; PPARα: peroxisome proliferatoractivated receptor-α; PGC1-α: PPAR-gamma coactivator 1-α; PTP1B: protein tyrosine phosphatase 1B; JNK: phosphorylation of c-Jun *N*-terminal kinase; ACE: angiotensin-converting enzyme; MAS: angiotensin (1–7) receptor.

## References

[1] International Diabetes Federation (IDF) (2015). IDF Diabetes Atlas.

[2] Pinhas-Hamiel O., Zeitler P. (2005). The global spread of type 2 diabetes mellitus in children and adolescents. J. Pediatr..

[3] Rando O.J., Simmons R.A. (2015). I’m eating for two: Parental dietary effects on offspring metabolism. Cell.

[4] Patel N., Pasupathy D., Poston L. (2015). Determining the consequences of maternal obesity for offspring health. Exp. Physiol..

[5] Malik V.S., Hu F.B. (2015). Fructose and cardiometabolic health: What the evidence from sugar-sweetened beverages tells us. J. Am. Coll. Cardiol..

[6] Marriott B.P., Cole N., Lee E. (2009). National estimates of dietary fructose intake increased from 1977 to 2004 in the United States. J. Nutr..

[7] Stephan B.C., Wells J.C., Brayne C., Albanese E., Siervo M. (2010). Increased fructose intake as a risk factor for dementia. J. Gerontol. Ser. A Biol. Sci. Méd. Sci..

[8] Hu F.B., Malik V.S. (2010). Sugar-sweetened beverages and risk of obesity and type 2 diabetes: Epidemiologic evidence. Physiol. Behav..

[9] Ogden C.L., Kit B.K., Carroll M.D., Park S. (2011). Consumption of sugar drinks in the United States, 2005–2008. NCHS Data Brief.

[10] Rosset R., Surowska A., Tappy L. (2016). Pathogenesis of cardiovascular and metabolic diseases: Are fructose-containing sugars more involved than other dietary calories?. Curr. Hypertens. Rep..

[11] Goran M.I., Dumke K., Bouret S.G., Kayser B., Walker R.W., Blumberg B. (2013). The obesogenic effect of high fructose exposure during early development. Nat. Rev. Endocrinol..

[12] Bray G.A., Nielsen S.J., Popkin B.M. (2004). Consumption of high-fructose corn syrup in beverages may play a role in the epidemic of obesity. Am. J. Clin. Nutr..

[13] Elliott S.S., Keim N.L., Stern J.S., Teff K., Havel P.J. (2002). Fructose, weight gain, and the insulin resistance syndrome. Am. J. Clin. Nutr..

[14] Basciano H., Federico L., Adeli K. (2005). Fructose, insulin resistance, and metabolic dyslipidemia. Nutr. Metab..

[15] Rippe J.M., Angelopoulos T.J. (2015). Fructose-containing sugars and cardiovascular disease. Adv. Nutr..

[16] Schulze M.B., Manson J.E., Ludwig D.S., Colditz G.A., Stampfer M.J., Willett W.C., Hu F.B. (2004). Sugar-sweetened beverages, weight gain, and incidence of type 2 diabetes in young and middle-aged women. JAMA.

[17] Fung T.T., Malik V., Rexrode K.M., Manson J.E., Willett W.C., Hu F.B. (2009). Sweetened beverage consumption and risk of coronary heart disease in women. Am. J. Clin. Nutr..

[18] Narain A., Kwok C.S., Mamas M.A. (2016). Soft drinks and sweetened beverages and the risk of cardiovascular disease and mortality: A systematic review and meta-analysis. Int. J. Clin. Pract..

[19] Brantsaeter A.L., Haugen M., Samuelsen S.O., Torjusen H., Trogstad L., Alexander J., Magnus P., Meltzer H.M. (2009). A dietary pattern characterized by high intake of vegetables, fruits, and vegetable oils is associated with reduced risk of preeclampsia in nulliparous pregnant Norwegian women. J. Nutr..

[20] Borgen I., Aamodt G., Harsem N., Haugen M., Meltzer H.M., Brantsaeter A.L. (2012). Maternal sugar consumption and risk of preeclampsia in nulliparous Norwegian women. Eur. J. Clin. Nutr..

[21] George G.C., Hanss-Nuss H., Milani T.J., Freeland-Graves J.H. (2005). Food choices of low-income women during pregnancy and postpartum. J. Am. Diet. Assoc..

[22] Englund-Ogge L., Brantsaeter A.L., Haugen M., Sengpiel V., Khatibi A., Myhre R., Myking S., Meltzer H.M., Kacerovsky M., Nilsen R.M. (2012). Association between intake of artificially sweetened and sugar-sweetened beverages and preterm delivery: A large prospective cohort study. Am. J. Clin. Nutr..

[23] Alzamendi A., Castrogiovanni D., Gaillard R.C., Spinedi E., Giovambattista A. (2010). Increased male offspring’s risk of metabolic-neuroendocrine dysfunction and overweight after fructose-rich diet intake by the lactating mother. Endocrinology.

[24] Vickers M.H., Clayton Z.E., Yap C., Sloboda D.M. (2011). Maternal fructose intake during pregnancy and lactation alters placental growth and leads to sex-specific changes in fetal and neonatal endocrine function. Endocrinology.

[25] Rawana S., Clark K., Zhong S., Buison A., Chackunkal S., Jen K.L. (1993). Low dose fructose ingestion during gestation and lactation affects carbohydrate metabolism in rat dams and their offspring. J. Nutr..

[26] Ching R.H., Yeung L.O., Tse I.M., Sit W.H., Li E.T. (2011). Supplementation of bitter melon to rats fed a high-fructose diet during gestation and lactation ameliorates fructose-induced dyslipidemia and hepatic oxidative stress in male offspring. J. Nutr..

[27] Saad A.F., Dickerson J., Kechichian T.B., Yin H., Gamble P., Salazar A., Patrikeev I., Motamedi M., Saade G.R., Costantine M.M. (2016). High-fructose diet in pregnancy leads to fetal programming of hypertension, insulin resistance, and obesity in adult offspring. Am. J. Obstet. Gynecol..

[28] Alzamendi A., Zubiria G., Moreno G., Portales A., Spinedi E., Giovambattista A. (2016). High risk of metabolic and adipose tissue dysfunctions in adult male progeny, due to prenatal and adulthood malnutrition induced by fructose rich diet. Nutrients.

[29] Gray C., Long S., Green C., Gardiner S.M., Craigon J., Gardner D.S. (2013). Maternal fructose and/or salt intake and reproductive outcome in the rat: Effects on growth, fertility, sex ratio, and birth order. Biol. Reprod..

[30] Gray C., Gardiner S.M., Elmes M., Gardner D.S. (2016). Excess maternal salt or fructose intake programmes sex-specific, stress- and fructose-sensitive hypertension in the offspring. Br. J. Nutr..

[31] Tain Y.L., Leu S., Wu K.L., Lee W.C., Chan J.Y. (2014). Melatonin prevents maternal fructose intake-induced programmed hypertension in the offspring: Roles of nitric oxide and arachidonic acid metabolites. J. Pineal Res..

[32] Hsu C.N., Wu K.L., Lee W.C., Leu S., Chan J.Y., Tain Y.L. (2016). Aliskiren administration during early postnatal life sex-specifically alleviates hypertension programmed by maternal high fructose consumption. Front. Physiol..

[33] Wallack L., Thornburg K. (2016). Developmental origins, epigenetics, and equity: Moving upstream. Matern. Child Health J..

[34] Douard V., Ferraris R.P. (2008). Regulation of the fructose transporter GLUT5 in health and disease. Am. J. Physiol. Endocrinol. Metab..

[35] David E.S., Cingari D.S., Ferraris R.P. (1995). Dietary induction of intestinal fructose absorption in weaning rats. Pediatr. Res..

